# Benign Endobronchial Tumors: A Clinicopathologic Review

**DOI:** 10.3389/fsurg.2021.644656

**Published:** 2021-03-05

**Authors:** Joshua E. Insler, Christopher W. Seder, Karina Furlan, Fatima Mir, Vijaya B. Reddy, Paolo Gattuso

**Affiliations:** ^1^Rush Medical College of Rush University Medical Center, Rush University Medical Center, Chicago, IL, United States; ^2^Department of Cardiovascular and Thoracic Surgery, Rush University Medical Center, Chicago, IL, United States; ^3^Department of Pathology, Rush University Medical Center, Chicago, IL, United States

**Keywords:** benign endobronchial lesion, endobronchial tumor, hamartoma, thoracic surgery, leiomyoma

## Abstract

**Purpose:** Benign endobronchial tumors are rare entities that can be difficult to diagnose because they often present with non-specific symptoms and vague radiographic findings. The current study reviews the clinical, radiologic and pathologic features, diagnosis, and treatment of patients with benign endobronchial tumors.

**Methods:** We examined the charts of all patients who presented with biopsy-proven benign endobronchial tumors at a tertiary-care academic medical center between 1993 and 2018. Pertinent clinicopathologic and radiologic data were analyzed, with particular attention paid to treatment modalities and mean overall patient survival.

**Results:** A total of 28 cases were identified. The most common benign neoplasm was hamartoma (37%), followed by lipoma (19%), squamous papilloma (11%), pleomorphic adenoma (7%), mucin gland adenoma (7%), papillary adenoma (3%), hemangioma (3%), neurofibroma (3%), leiomyoma (3%), and papillomatosis (3%). Cough (58%), shortness of breath (44%), and hemoptysis (15%) were the most frequent presentations. Most cases demonstrated well-defined submucosal or pedunculated endobronchial lesions with segmental pneumonia or atelectasis on imaging. Histologic diagnosis was obtained by endobronchial resection in 43% of patients, thoracoscopic lobectomy in 36%, endobronchial biopsy in 18%, and thoracoscopic wedge resections in 3%. All procedures were performed with no intraoperative or in-hospital deaths (mean overall survival: 20.2 years).

**Conclusion:** Benign endobronchial tumors typically present as well-defined submucosal and/or pedunculated lesions, and may lead to post-obstructive complications. Endobronchial resection is the preferred strategy for diagnosis and treatment of these tumors.

## Introduction

Benign endobronchial tumors are particularly uncommon clinical entities, with significant variability in etiology and presentation (1). They are typically slow-growing, and frequently present with symptoms of bronchial obstruction and compression of local structures, leading to cough, wheezing, or chest discomfort (2). Radiographic manifestations of these lesions are often vague, including atelectasis, bronchiectasis, recurrent pneumonia, or mediastinal shift, making them difficult to distinguish from malignant tumors (2, 3).

Many conditions can lead to endobronchial lesions, including those of neoplastic, inflammatory, and iatrogenic origin (4). Although individual case reports and small case series have described specific benign endobronchial tumors, few reports have comprehensively outlined the spectrum of presentation, diagnosis, and treatment of benign endobronchial lesions (3, 4) The aim of the present study was to provide an overview of the clinicopathologic and radiologic features, modes of diagnosis and treatments, and mean overall patient survival for patients presenting with biopsy-proven benign endobronchial tumors at a single, tertiary-care, academic medical center.

## Methods

An institutional database was queried to identify patients with biopsy-proven lesions of the tracheobronchial tree between 1993 and 2018 at Rush University Medical Center and perform a retrospective cohort study. Using our institution's electronic medical record (EMR), patient charts were used to collect and report demographic, clinical, histologic, radiographic, and mean overall patient survival.

Need for individual consent was waived, and Institutional Review Board approval was obtained prior to initiation of this study. Available charts of all patients who had presented with biopsy-proven benign endobronchial lesions at our institution between the years of 1993 and 2018 were queried and comprehensively examined by searching both the EMR and patient records from the departments of thoracic surgery and pathology. Patient charts from prior to our institution's transition to the EMR which were not successfully transferred from paper to EMR were excluded from the present study. This search process yielded a total of 28 patient charts.

All surgical patients followed-up in the outpatient clinic at both 2 weeks and 6 months following discharge, and were encouraged to return to the clinic if they became symptomatic or for any additional questions or concerns. Non-surgical patients were instructed to return to the outpatient clinic after 6 months and/or if their symptoms worsened. No patients experienced recurrence of the lesions or malignant transformation of their original lesions after diagnosis and treatment. Two patients (7%) had died at the time of data collection.

## Results

**Figure 1:**
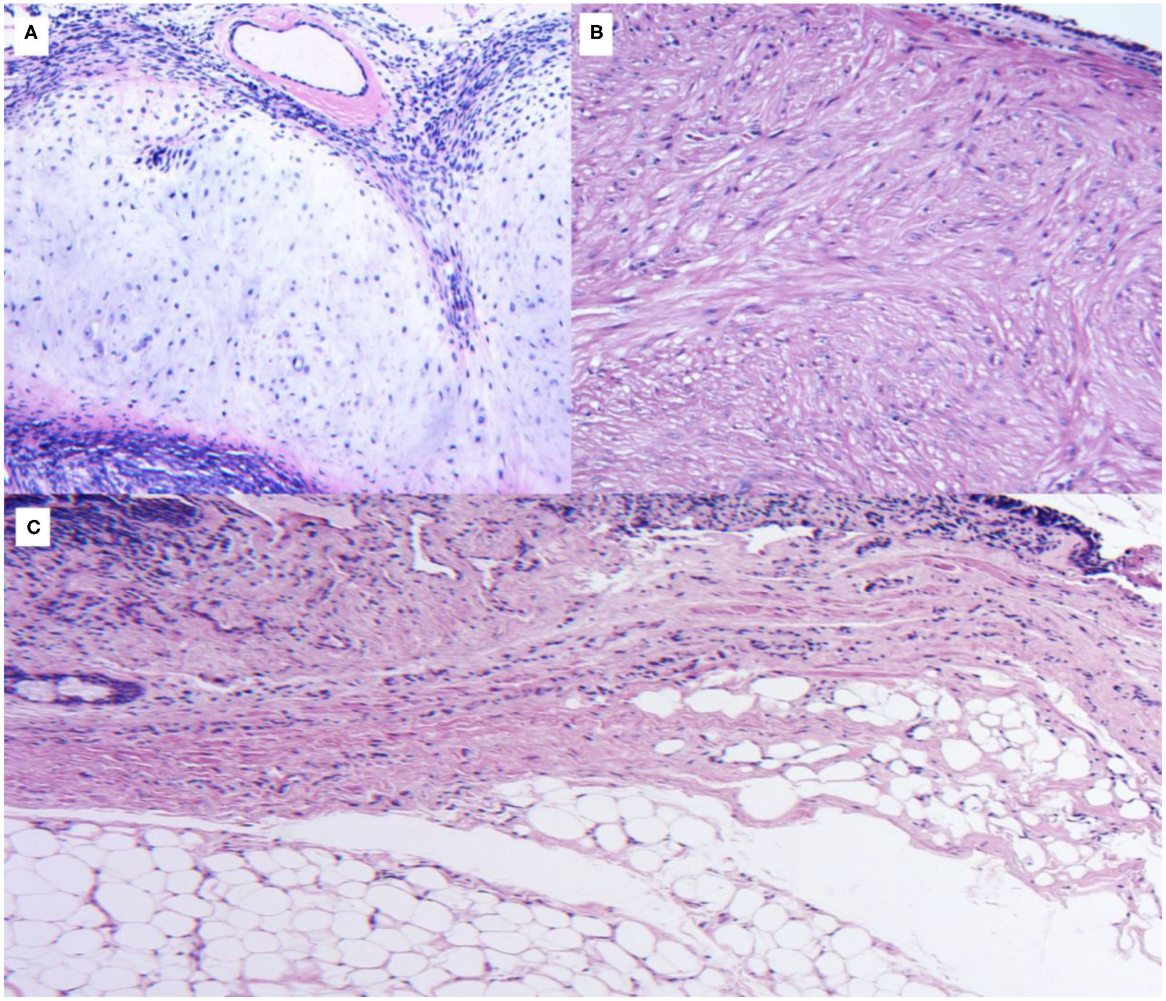
**(A)** Histologic sections of a mainstem bronchus lesion show a proliferation of mature cartilaginous tissue surrounded by spindle cells, consistent with a hamartoma. No mitotic figures or necrosis are appreciated, confirming the benign nature of the lesion. **(B)** Histologic section of an intrabronchial lesion. A thin lining of ciliated cuboidal respiratory epithelium is identified. A fascicular proliferation of smooth muscle bundles with eosinophilic and occasional fibrillar cytoplasm is seen within the tumoral lesion, finding consistent with a benign leiomyoma. **(C)** Histologic section of a common intrabronchial lesion recovered in our database. A lining is observed surrounding the lesion, compatible with respiratory epithelium. The lesion is composed of mature fibroadipose tissue without evidence of atypia or necrosis, consistent with lipoma.

A total of 28 cases of benign endobronchial neoplasms were identified (Table 1) that met study inclusion criteria. Hamartomas were the most common lesion, identified in 10 patients (Figure 1A). Lipomas were identified in 4 of patients (Figure 1C), and squamous papilloma in 3 of patients. The remaining benign neoplasms, including and leiomyoma (Figure 1B) and pleomorphic adenoma, were each identified in <3 patients.

**Table 1:** Incidence and presentation of benign endobronchial lesions treated between 1993 and 2018.

**Histology**	***n***	**Incidence**	**Male**	**Race**	**Smoker**	**Respiratory symptoms**
Hamartoma	11	39%	7 (63%)	8 Caucasian, 3 African American,	3 (27%)	10 (9%)
Lipoma	5	18%	1 (20%)	4 Caucasian, 1 Hispanic	2 (40%)	2 (40%)
Squamous papilloma	3	11%	2 (67%)	3 Caucasian	2 (67%)	1 (33%)
Pleomorphic adenoma	2	7%	1 (50%)	1 Caucasian, 1 Hispanic	0 (0%)	0 (0%)
Mucin gland adenoma	2	7%	0 (0%)	2 Caucasian	1 (50%)	0 (0%)
Papillary adenoma	1	3%	0 (0%)	1 Caucasian	0 (0%)	0 (0%)
Hemangioma	1	3%	0 (0%)	1 Caucasian	0 (0%)	0 (0%)
Neurofibroma	1	3%	1 (100%)	1 Caucasian	0 (0%)	0 (0%)
Leiomyoma	1	3%	0 (0%)	1 African American	0 (0%)	0 (0%)
Papillomatosis	1	3%	1 (100%)	1 Caucasian	0 (0%)	0 (0%)
*Total*	28	100%	13 (46%)	22 Caucasian, 4 African American, 2 Hispanic	8 (29%)	12 (43%)

Overall, 13 patients were male and 15 were female. The age ranged between 51 and 72 years, with a mean age of 58.4 years. Mean body mass index for all patients (BMI) was 28.9. Asthma and chronic obstructive pulmonary disease (COPD) were the most common comorbidities, present in 10 and 12 patients, respectively. Males accounted for the majority of endobronchial hamartomas; there was not a significant difference in sex for the other neoplasms. Twenty nine percent of patients were smokers; 3 with hamartomas and 2 with lipomas. Cough was the most commonly reported symptom, present in 58% of patients; cough and shortness of breath were concurrently present in 8 patients (29%). The next most common symptom, dyspnea, was reported in 44% of patients. Hemoptysis was reported in 4 patients (15%). Wheezing was reported in 6 patients (21%) and 10 patients (36%) did not present with respiratory symptoms at the time of diagnosis, and instead presented with fatigue, muscle weakness, or numbness.

**Figure 2:**
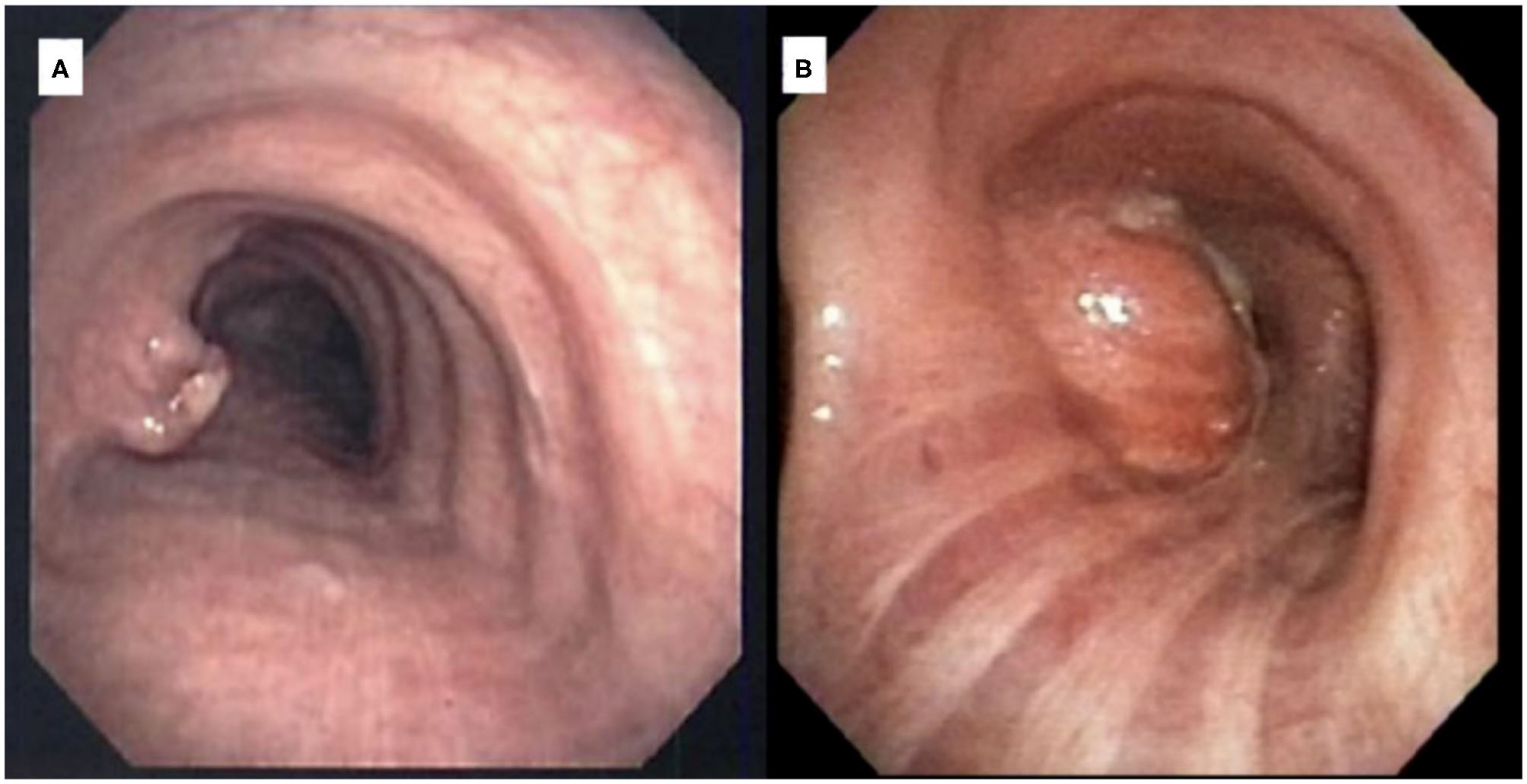
Bronchoscopic imaging demonstrating **(A)** hamartoma and **(B)** leiomyoma.

**Figure 3:**
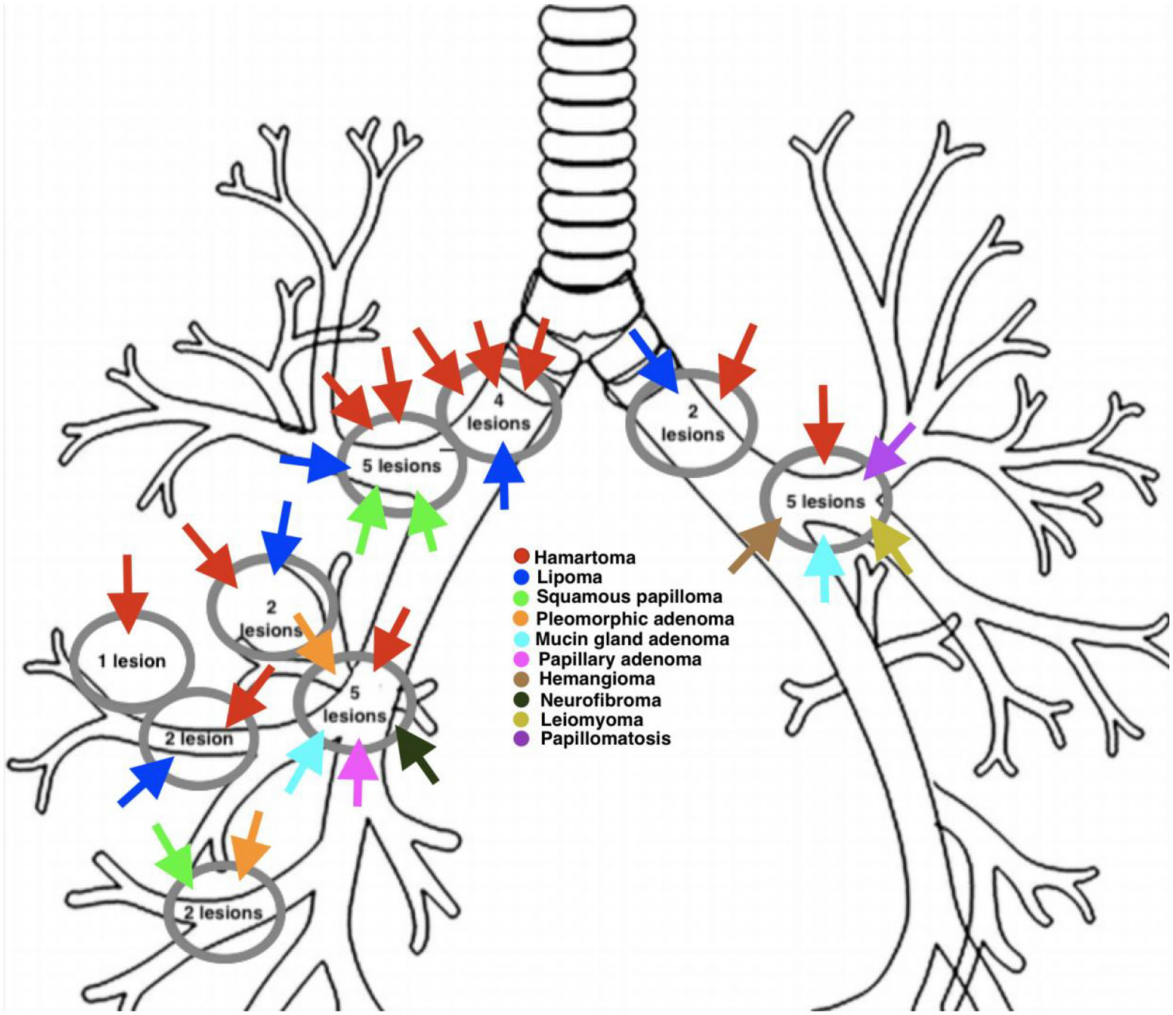
Anatomic locations of benign endobronchial lesions of patients diagnosed and treated at Rush University Medical Center between 1993 and 2018.

The mean size of patients' lesions on computed tomography (CT) imaging was 1.1 × 0.9 × 0.5 cm. All cases demonstrated well-defined lesions on flexible fiberoptic bronchoscopy. For a majority of patients, bronchoscopy revealed well-defined submucosal endobronchial lesions (59%), with the rest demonstrating well-defined pedunculated endobronchial lesions (41%) (Figure 2). Figure 3 visually depicts the anatomic locations of patients' lesions. Bronchoscopy revealed 5 lesions to be located in the right upper lobe takeoff, 5 in the left upper lobe takeoff, 4 in the right lateral wall of the carina, 2 in the left lateral wall of the carina, 5 in the bronchus intermedius, 4 among right segmental bronchi, and 3 among right subsegmental bronchi.

A majority of cases demonstrated submucosal or pedunculated endobronchial lesions associated; a minority of cases demonstrated exophytic endobronchial lesions described as oval, spherical, or polypoid in shape with lobulated borders. Imaging revealed segmental pneumonia in 4 cases (24%) and atelectasis in 3 cases (18%).

Interventions for all patients are presented in Table 2. Endobronchial ultrasound with core needle biopsy was performed for diagnosis in 18% of patients in the present study. Twenty patients underwent core needle biopsies; 6 patients underwent forceps biopsies; 2 patients underwent fine needle aspirations. Endobronchial excision using an Nd YAG laser was performed in 43% of patients; lobectomy was performed in 36% of patients, with 8 patients requiring video-assisted thoracoscopic surgery (VATS) and 2 patients requiring thoracotomy; VATS wedge resections were performed in 3% of patients. Of those patients undergoing lobectomy, only one patient required bronchoplasty; no patients required additional parenchymal sparing resection. One patient experienced a urinary tract infection postoperatively (5%); there were otherwise no complications to report. No patients who underwent endobronchial excision, lobectomy, or wedge resection required repeat endobronchial resection. All procedures were performed with no in-hospital deaths.

**Table 2:** Management and postoperative complications of 28 patients with benign endobronchial lesions.

**Procedure**	**Number of patients**	**Percentage**	**Complications**
Endobronchial excision	12	43%	2 patients, atelectasis
Thoracoscopic lobectomy	10	36%	2 patients, pneumothorax
Wedge resection	1	3%	1 patient, pneumothorax
Biopsy only	5	18%	5 patients, no complications
Total	28	100%	100%

## Discussion

To our knowledge, the current study is one of the first to report the spectrum of clinicopathologic presentation of benign endobronchial tumors. It adds to the existing literature by providing an overview of the most common subtypes, clinical characteristics, and management of these lesions. Although benign, these tumors can lead to complications related to obstruction, therefore timely diagnosis is crucial to institute appropriate management.

The benign endobronchial tumors in the current study were predominantly of mesenchymal origin. The most common mesenchymal lesion, hamartoma, was found in 11 patients (39%), with more than half of cases occurring in men. These findings, along with the relatively lower occurrence of other mesenchymal lesions such as lipoma and neurofibroma, are similar to prior series (5, 6). Tumors of submucosal glands and of surface epithelial origin, including mucinous gland adenoma, papillary adenoma, pleomorphic adenoma, and squamous papilloma, were among the most infrequently occurring lesions, further corroborating data from both individual case series (7, 8) and literature reviews (9). Histopathologic diagnosis of an endobronchial tumor is not that difficult; however, diagnosis on small endobronchial biopsies may be challenging, especially in the case of papillary adenoma and mucous gland adenoma where morphologic findings could mimic low grade adenocarcinoma. Cough was the most commonly reported symptom (53% of patients) followed by dyspnea (47% of patients), which are reported in greater frequency in comparison to the findings of previously reported case series (10); this may be due to minor variance in the anatomic locations of each patient's lesion and the degree to which it presented with obstructive symptoms.

With recent advances in bronchoscopic instrumentation and navigation, the first-line approach in the diagnosis of benign endobronchial tumors is most often endobronchial imaging and biopsy, which serves to inform the surgeon of the histology and location of the tumor (11). This may be done either with or without endoscopic ultrasound for diagnostic purposes and surgical planning. If the tumor is small, it may be excised during initial bronchoscopic evaluation using a snare or forceps (11). Debulking may be achieved via a variety of different methods during bronchoscopy, including electrocautery snare, Nd YAG laser, and microwave ablation (11, 12). In cases of large, broad based or vascular tumors, where there is a higher risk of hemorrhage or airway loss, the use of a rigid bronchoscope for debulking and airway control may be more appropriate (11, 12). Surgical management with minimally-invasive parenchyma-sparing procedures should be prioritized and performed when possible. Preoperative evaluation of histology, pathologic staging, and pulmonary function should be accomplished during initial workup for risk stratification.

In cases where debulking of a benign tumor is required, our preferred approach includes initial flexible bronchoscopy to evaluate the location and appearance of the tumor. If the tumor is in the trachea or proximal main stem airways, we will commonly do so by coring it out with a rigid bronchoscope and achieving hemostasis using Nd YAG laser ablation. In this series lesions, all were able to be debulked without rigid bronchoscopy. In cases where the lesion is more distal in the bronchial tree, but visible with white light bronchoscopy, we will ablate using Nd YAG laser and debride the tumor using cold forceps. This technique does not vary based on histology, but rather on location of the tumor.

While none of the patients in the present study experienced malignant degeneration of their original lesions, transformation of benign hamartoma, lipoma, and squamous papilloma into malignant chondrosarcoma, liposarcoma, and papillary squamous cell carcinoma has been reported and are very rare complications of these typically benign tumors (13–15). For patients who adhere to follow-up with conservative observation in the outpatient clinic, this risk has been noted to be low—and malignancy, if present, may be diagnosed early on (15).

The primary limitation of the current study is the relatively small sample size, which does not allow for correlation between age, gender, diagnosis, symptomatology, and presentation at the time of diagnosis. Moreover, because this study spanned a time of transition from paper charts and an EMR, some imaging studies were unavailable. In spite of this, the external validity of the current study is high given the fact that we report “real world” data.

In conclusion, the current study provides an overview of the clinical and radiologic features, diagnosis, and treatment of patients with benign endobronchial tumors. These tumors typically present as well-defined submucosal or pedunculated tumors, and have the potential to cause post-obstruction complications. Healthcare providers should include these lesions in the differential diagnosis of lung neoplasms, as they are frequently indistinguishable from malignant lung tumors, radiologically and clinically.

## Author Contributions

JI composed the manuscript and developed data presentation. KF, FM, VR, and PG contributed equally to the revision and review process of the manuscript. All authors contributed to the article and approved the submitted version.

## Conflict of Interest

The authors declare that the research was conducted in the absence of any commercial or financial relationships that could be construed as a potential conflict of interest.
